# LncRNA CRNDE promotes hepatoma cell proliferation by regulating the metabolic reprogramming of M2 macrophages via ERK pathway

**DOI:** 10.1186/s12935-024-03380-8

**Published:** 2024-05-31

**Authors:** Chao Lin, Tao Jiang, Changyong E, Lun Wang, Tong Chen, Xia Wang, Yien Xiang

**Affiliations:** 1 Hepatobiliary and Pancreatic Surgery, China-Japan Union Hospital of Jilin University, Changchun, China; 2 Gastrointestinal Surgery, Affiliated Hospital of Zunyi Medical University, Zunyi, China; 3 Gastrointestinal Surgery, China-Japan Union Hospital of Jilin University, Changchun, China; 4 General Surgery, China-Japan Union Hospital of Jilin University, Changchun, China; 5 Hepatobiliary and Pancreatic Surgery, the Second Hospital of Jilin University, Changchun, China

**Keywords:** CRNDE, Macrophage, Metabolic reprogramming, Hepatocellular carcinoma

## Abstract

**Background:**

LncRNA colorectal neoplasia differentially expressed (CRNDE) was found to be an important regulator in many cancers. This project focuses on the function of CRNDE on macrophage metabolic reprogramming and Hepatocellular carcinoma (HCC).

**Method:**

qRT-PCR and Immunofluorescence were used to analyze Arg-1, IL-10, CD163, CCL-18, CD206, and CRNDE expression in HCC tissues and macrophages. Western Blotting was used to analyze ERK and p-ERK expression. Edu assay, transwell assay and xenograft experiments were carried out to study cell viability, migrated and invasive capability. Immunohistochemical staining was used to evaluate Ki67 expression. A liquid chromatography-tandem mass spectrometry (LC-MS/MS) was performed for macrophages metabolites analysis.

**Results:**

Arg-1, IL-10, CD163, CD206, and CRNDE were significantly up-regulated in HCC tissues, M2 macrophage and M0 macrophage with CRNDE overexpressed (OV-CRNDE-M0), which downregulated in M0 macrophage with CRNDE knockdown (sh-CRNDE-M0). The conditioned medium (CM) of M2 cells and OV-CRNDE-M0 cells promoted cell viability, invasion, and migration of HCC cells, the effect was reversed by sh-CRNDE-M0 cells CM. OV-CRNDE-M0 cells promoted tumor growth, Ki67 and CD206 expression in xenograft model. 61 metabolites were detected, of which 18 metabolites changed significantly in OV-CRNDE-M0 group compared to M0 group, with 9 upregulated and 9 downregulated. KEGG analysis showed the enrichment pathways were biosynthesis, glyoxylate and dicarboxylate metabolism. SMPDB analysis showed the enrichment pathways were hypoacetylaspartia, canavan disease, and aspartate metabolism.

**Conclusion:**

CRNDE regulated the metabolic reprogramming of M2 macrophage via ERK pathway, which thereby contributed to HCC proliferation, migration, and invasion.

**Supplementary Information:**

The online version contains supplementary material available at 10.1186/s12935-024-03380-8.

## Background

Hepatocellular carcinoma (HCC) had the highest incidence in primary liver malignancy [1], with 906,000 new patients and 830,000 death toll worldwide in 2020, and ranked the third most frequent cause of cancer death around the world [2, 3]. Surgery and transplantation were only suitable for early-stage patient treatment [4]. Drug resistance and high tumor recurrence rates make the treatment and management of HCC complicated [5]. Therefore, exploring the pathogenesis and find new therapeutic targets was still meaningful for HCC.

Macrophage was a pivotal immune cell that participated and functioned in tumor angiogenesis and progress [6]. Notably, macrophages go through different metabolic ways, polarizing into M1 (classically activated) and M2 (alternatively activated) [7, 8] phenotypes in response to the extrinsic environmental signals. Macrophages within the tumor, are a critical regulator of tumor development that could be metabolic reprogrammed by inflammatory mediators in the tumor microenvironment (TME) [9]. Furthermore, Macrophages gained lots of energy and metabolic intermediates to undergo phenotypic alterations and functional modifications in the TME through metabolic reprogramming [10]. Therefore, controlling macrophages metabolic reprogramming may be new therapeutic approaches for cancer.

Dong et al. has reported that long noncoding RNAs (lncRNAs) engaged in the M2 polarization and metabolic of both cancer cells and macrophages [12, 13]. The lncRNA colorectal neoplasia differentially expressed (lncRNA CRNDE), a 1910-nt lncRNA, has been found to be involved in osteoarthritis [15], delayed encephalopathy [16], and tumor progress [17, 18]. CRNDE was elevated in tumor tissues and macrophages of patients with gastric carcinoma [19], and was identified to be oncogenic RNA in cancers [20]. CRNDE promoted HCC development through increasing SIX1 expression [21]. It also promoted tumor angiogenesis by upregulation of M2 macrophages markers [22]. However, the effect of CRNDE on macrophage metabolic reprogramming has not been studied.

This study was designed to investigate the role of CRNDE in regulation of macrophage metabolic reprogramming and polarization, as well as the underlying mechanism, which providing new ideas and strategies for HCC therapy.

## Materials and methods

### Patient samples

HCC tissues samples have been taken for qRT-PCR and immunofluorescence staining (IFC). All subjects have completed the informed consent.The research involving clinical samples was approved by the Ethics Committee of the China-Japan Union Hospital of Jilin University (Date 7.3.2023/No.2,023,070,309).

### Cell culture

Huh7, Hep3B, and human monocytic cell line (THP-1 cells) were obtained from iCell Bioscience Inc (Shanghai, China). Huh7 cells and THP-1 cells were maintained using the method reported previously [23, 24]. Hep3B cells were maintained in Minimum Essential Media (MEM, Gibco) containing 10% FBS, 1%P/S, 1% L-Glutamine, 1%NEAA and 1% sodium pyruvate (iCell Bioscience Inc, Shanghai, China).

### THP-1 cells differentiated

For differentiation to naïve macrophages (M0) cells, THP-1 cells were incubated with 150 nM phorbol-12-myristate-13-acetate (PMA, Solarbio, Beijing, China) for 24 h, and then were cultured with fresh medium for 24 h.

For M2 polarization, THP-1 cells were incubated with 150 nM PMA for 24 h, then stimulated with 20 ng/mL IL-4, and 20 ng/mL IL-13 (Sino Biological, China) for another 48 h. qPCR was used to detect the relative expression of M2 macrophage markers Arg-1, CCL-18 and IL-10.

### RNA extraction and qPCR

Total RNAs were extracted from cells or tumor tissue and paracancer tissue of patients and mice by using RNAiso Plus (Cwbio, China), followed by cDNA synthesis with reverse transcription kit (TransGen Biotech, China). The experiment was operated on LightCycler 96 Real time PCR (Roche, Switzerland). All premiers were derived from Sangon Biotech (Shanghai, China) and showed in Table 1. The expression levels of LncRNA-CRNDE, Arg-1, CCL-18 and IL-10 were normalized based on the expression of GAPDH.

**Table 1:** Primer sequence

Primer	Sequence (5’-3’)
LncRNA-CRNDE-F	TCCTCTGTCCACGCCTGTTC
LncRNA-CRNDE-R	CACCTCCAAGGGCTCTACTCC
Arg-1-F	ACCTGCCCTTTGCTGACATC
Arg-1-R	ACCAGGCTGATTCTTCCGTT
CCL-18-F	ACCTCCTGGCAGATTCCACA
CCL-18-R	TGCCGGCCTCTCTTGGTTA
IL-10-F	TGCCAAGCCTTGTCTGAGAT
IL-10-R	GAGTTCACATGCGCCTTGAT
GAPDH-F	AATCCCATCACCATCTTCCA
GAPDH-R	AAATGAGCCCCAGCCTTCT

### Immunofluorescence

THP-1 cells (1 × 10^6^/mL) were seeded on glass coverslips in 6-well plate, and then blocked with 3% BSA for 30 min. After that, cells were incubated with CD206 monoclonal antibody at 4 °C overnight (dilution 1:300, Wuhan Sanying, China). After washing with PBS for 3 times, cells were incubated with fluorescent secondary antibody, following with DAPI staining. The cells were centrifuged and re-suspended in PBS, the cell suspension was dropped onto a slide, and a cover slip was added.

The tissue samples of patients and mice were prepared into paraffin sections and incubated in 10% goat serum for 30 min. The primary antibody was incubated at 4℃ overnight and washed with PBS solution. After that, CD206 (dilution 1:300, Wuhan Sanying, China) was added and incubated at 37℃ for 1 h. DAPI was used to restain the nucleus.

The slides were examined using XD202 microscope (Ningbo Yongxin optics CO., LTD. China).

### Cell transfection

The silencing RNA targeting lnc CRNDE (sh-RNA-CRNDE), were synthesized by Gene-Pharma (Shanghai, China). PcDNA3.1-CRNDE was synthesized by GENERAL BIOL (Anhui, China). Sh-RNA-CRNDE and pcDNA3.1-CRNDE were transfected into cells using Lipofectamine 2000 (Invitrogen, China), and transfection efficiency was determined by qPCR analysis.

### Edu assay

Cells proliferative potential was measured by A 5-ethynyl-20-deoxyuridine (EdU) assay kit (Beyotime Biotechnology, China). The conditioned medium (CM) of M0, M2, OV-CRNDE-M0, and sh-CRNDE-M0 were collected and filtered. Hep3B and Huh-7 cells (1 × 10^5^ cells) were seeded in 24-well plates and cultured overnight. Then cells were treated with CM of M0, M2, OV-CRNDE-M0, and sh-CRNDE-M0 for 24 h. Following the instructions, the cells were incubated with 10 µM EdU for 2 h. After fixed and permeabilized, cells were captured using XD202 microscope (Ningbo Yongxin optics CO., LTD. China).

### Migration and invasion assays

The CM of M2, OV-CRNDE-M0, and sh-CRNDE-M0 were collected, filtered, and used in transwell assay. HCC cells with serum-free medium were placed into the upper chamber, CM of M2, OV-CRNDE-M0, and sh-CRNDE-M0 were added into the bottom plates. After incubation for 24 h, cells on the upside were erased, and the underlying ones were fixed and stained with 1% crystal violet (Sangon Biotech, China). Then cells were calculated under 6 fields of view.

### Western blot

The experiment was divided into four groups, M0 cells, M2 cells, OV-CRNDE-M0 cells, and sh-CRNDE-M0 cells were treated with 5 µM ERK inhibitor (MCE, China). Cells were subsequently collected, washed with PBS, lysed in RIPA lysis buffer (Beyotime Biotechnology, China) containing PMSF solution (Beyotime Biotechnology, China). After electrophoresis with 15% polyacrylamide, samples were transferred, and then blocked with 5% BSA for 2 h. PVDF membranes were incubated with GAPDH (Servicebio, China), ERK (Dilution ratio: 1:1000) and p-ERK (Dilution ratio: 1:500) (Lingsi Biotechnology, China) monoclonal antibodies diluted in TBST. After washed with TBST for 4 times, the membranes were incubated with secondary antibody solution (Servicebio, China). The membranes were visualized by ECL solution. The expression levels of ERK and p-ERK were normalized based on the expression of GAPDH.

### 10 xenograft mice model

BALB/c nude mice (6–8 weeks) were segregated into 3 groups, Hep3B group, Hep3B + M0 group, and Hep3B + OV-CRNDE-M0 group. In the Hep3B group, Hep3B cells (4 × 10^5^/mL) were diluted in 100 µL of PBS. In the Hep3B + M0 group, Hep3B cells (4 × 10^5^/mL) and M0 cells (1 × 10^5^/mL) were diluted in 100 µL of PBS. In the Hep3B + OV-CRNDE-M0 group, Hep3B cells (4 × 10^5^/mL) and OV-CRNDE M0 cells (1 × 10^5^/mL) were diluted in 100 µL of PBS. All cells were subcutaneously injected to the left flank of the mice. Tumor sizes were measured every day. At 7-days following injection, mice were sacrificed and tumor tissue was obtained. Tumor volume was calculated with the formula: tumor volume (mm^3^) = 0.5×width (mm)^2^ × length (mm). The animal experiment was approved by the Ethics Committee of the First Hospital of Jilin University (Date 9.11.2023/No.0651).

### Immunohistochemistry

Proliferation index Ki67 were determined using immunohistochemical staining. Paraffin-embedded samples were blocked with 1% BSA for 30 min, and incubated with Ki67 antibody (Linges Biotechnology, China).

### 12 metabolite extraction and LC–MS/MS analysis

M0, M2, and OV-CRNDE-M0 cells pellets were resuspended in 100 µL ultrapure water. 200 µL 80% chilled methanol was added into cell suspension, the mixture was vortexed, frozen, and thawed for 3 times. After centrifugation for 10 min with 13,000 g at 4 ◦C, the supernatant was put into a new container and used for LC–MS/MS analysis.

Liquid chromatography was performed on an Ultra Performance Liquid Chromatography (UPLC, Waters ACQUITY H-Class D). The ACQUITY UPLC BEH Amide column (100 × 2.1 mm, 1.7 μm, Waters) was used with the following LC parameters: column temperature, 40 °C; flow rate, 0.4 mL/min. The LC solvents were as follows: Solvent A, ultrapure water (10mM ammonium acetate 0.3% ammonia water), Solvent B, 90% acetonitrile/water. The programmed gradients were as follows: for t = 0–1.2 min, 5%A and 95%B; for t = 8 min, 30%A and 70%B; for t = 9–11 min, 50%A and 50%B; for t = 11.1–15 min, A/B = 5% and 95%. Mass spectrometric analyses were performed with a Tandem Mass Spectrometry (Applied Biosystems, UK). Data acquisition and processing were achieved by Analyst 1.6.3 software and MultiQuant 3.0.3 software.

### Statistical analysis

The data in this work were presented as means ± S. D, and analyzed by SPSS 22.0 software. The statistical differences among different groups were analyzed by Student’s t-test or one-way ANOVA. *P* < 0.05 were considered to be statistically significant.

Principal component analysis (PCA) was performed to reveal the data structure.

## Results

### CRNDE was overexpressed in liver cancer

**Fig. 1:**
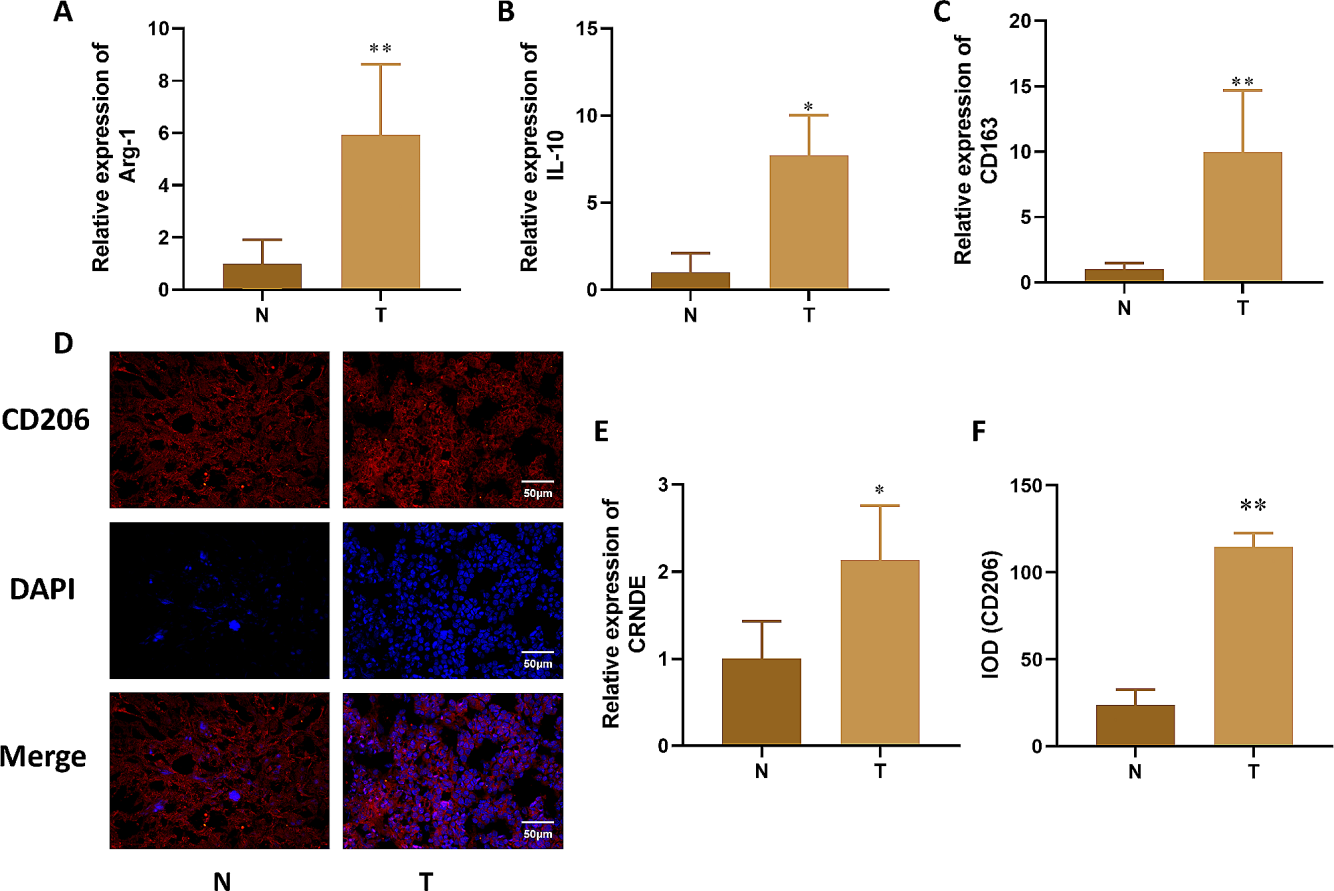
The expression of Arg-1, IL-10, CD163 and CRNDE in liver cancer tissue. **A**-**C**. Expression changes of M2 macrophage specific markers (CD163, Arg-1, IL-10) detected by qRT PCR in liver cancer tissue. **D**. Immunofluorescence detection of M2 surface antigen (CD206) expression changes in liver cancer tissue. **E**. qPCR was used to detect the expression of CRNDE in tissues. **F**. Quantitative of immunofluorescence staining

We first analyzed M2 macrophage markers, such as Arg-1, IL-10, and CD163 in tumor and paracancer tissues. The mRNA expression of Arg-1, IL-10, and CD163 were markly up-regulated in tumor samples compared to paracancer tissue samples (Fig. 1A-C). IFC staining results showed that CD206 was higher expressed in tumor samples than paracancer tissue samples (*P*<0.05) (Fig. 1D and F). Furthermore, the CRNDE mRNA was up-regulated in tumor samples compared with normal tissues (Fig. 1E).

### Overexpression of CRNDE in M0 cells induced M2 polarization

**Fig. 2:**
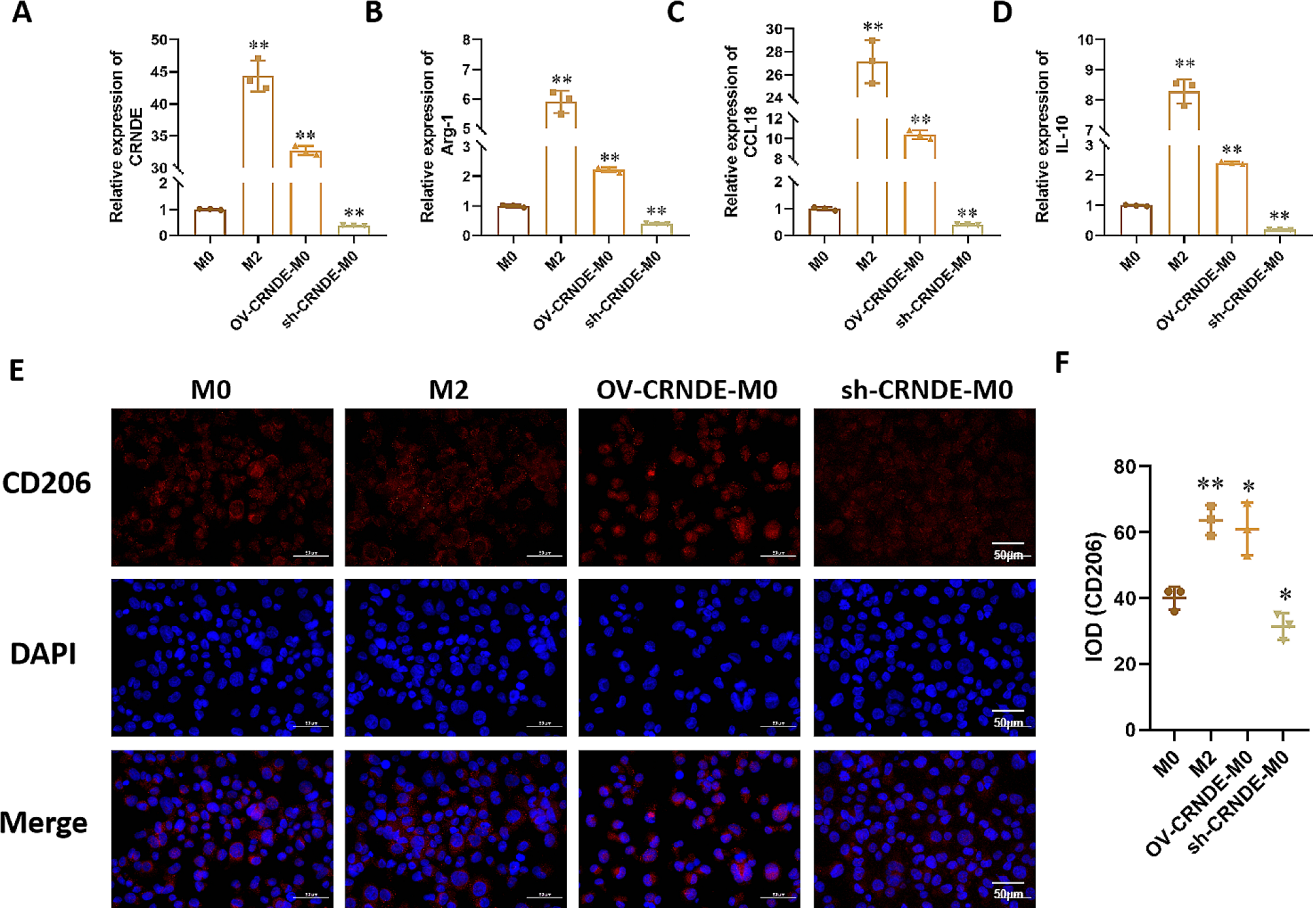
The effect of CRNDE on M2 polarization. **A**-**D**. Relative expression of CRNDE (**A**), Arg-1(**B**), CCL18 (**C**), IL-18 (**D**) in M0 cells, M2 cells, M0 cells transfected with CRNDE overexpression plasmid (OV-CRNDE-M0), and M0 cells transfected with CRNDE shRNA (sh-CRNDE-M0). G. CD206 expression by immunofluorescence staining. Compared with M0 group, *P < 0.05, **P < 0.01

According to the best inhibitory effect, sh-CRNDE 687 has the best inhibition efficiency (*P*<0.05). The expression of CRNDE was significantly up-regulated (*P*<0.05), when treated M0 cells with pcDNA3.1, (Supplementary Figure). Compared with M0 group, the CRNDE expression was significantly up-regulated in M2 group and OV-CRNDE-M0 group, while the expression in the sh-CRNDE-M0 group was lower than M0 group (*P*<0.05) (Fig. 2A). We then analyzed the expression levels of M2 macrophage markers, including Arg-1, CCL-18 and IL-10. Compared with M0 group, the expression of these 3 markers were up-regulated in M2 group and in OV-CRNDE-M0 group, which were down-regulated in sh-CRNDE-M0 group (*P*<0.05) (Fig. 2B-D). Immunofluorescent staining was performed to detect CD206 expression. Compared with M0 group, the CD206 expression were higher in M2 group and OV-CRNDE-M0 group, which was lower in sh-CRNDE-M0 group (*P*<0.05) (Fig. 2E-F).

### Effects of CRNDE on tumor cell proliferation and metastasis in vitro

**Fig. 3:**
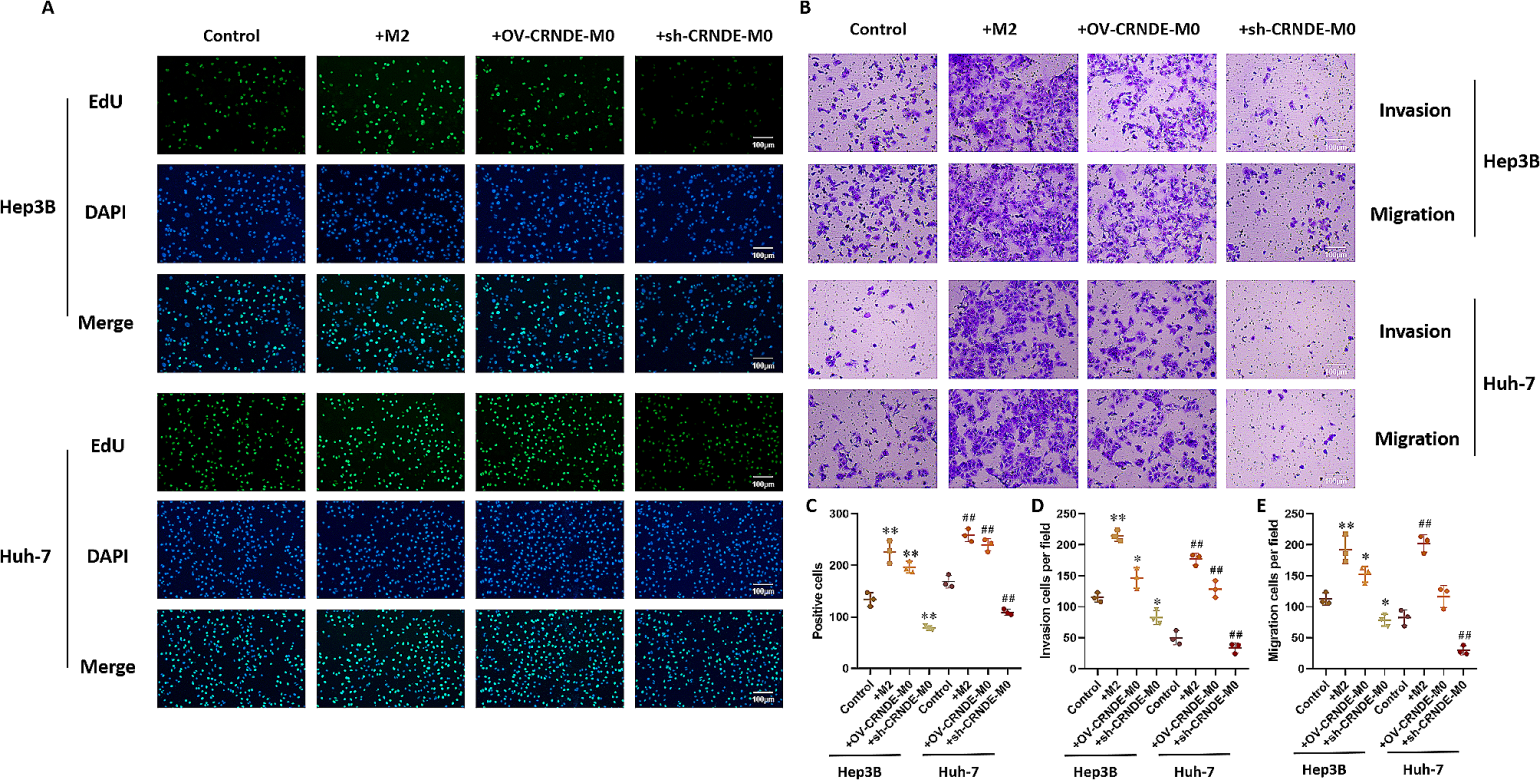
The effect of CRNDE in M0 cells on the proliferation and migration of liver cancer cells. **A**. Cells proliferative property was analyzed by the EdU assays. **B**. The migration/invasion of Hep3B cells and Huh-7 cells were detected by Transwell assay (200×). **C**. The quantitative analysis of EdU assay. **D**. The quantitative analysis of invasion assay. **E**. The quantitative analysis of migration assay. Compared with M0 group, *P < 0.05, **P < 0.01

To investigate the effects of CRNDE on Hep3B cells and Huh-7 cells proliferation and metastasis, the conditioned medium (CM) of M0, M2, OV-CRNDE-M0, and sh-CRNDE-M0 were collected. EdU assays showed that compared with M0 group, there were more positive cells in M2 group and OV-CRNDE-M0 group, while there were fewer positive cells in sh-CRNDE-M0 group (*P*<0.05) (Fig. 3A and C). Transwell assay demonstrated that M2 CM and OV-CRNDE-M0 CM promoted cells migration and invasion compared with M0 CM, while sh-CRNDE-M0 CM inhibited cancer cells migration and invasion (*P*<0.05) (Fig. 3B and D-E).

### Effects of CRNDE in M0 cells promoted xenograft growth

**Fig. 4:**
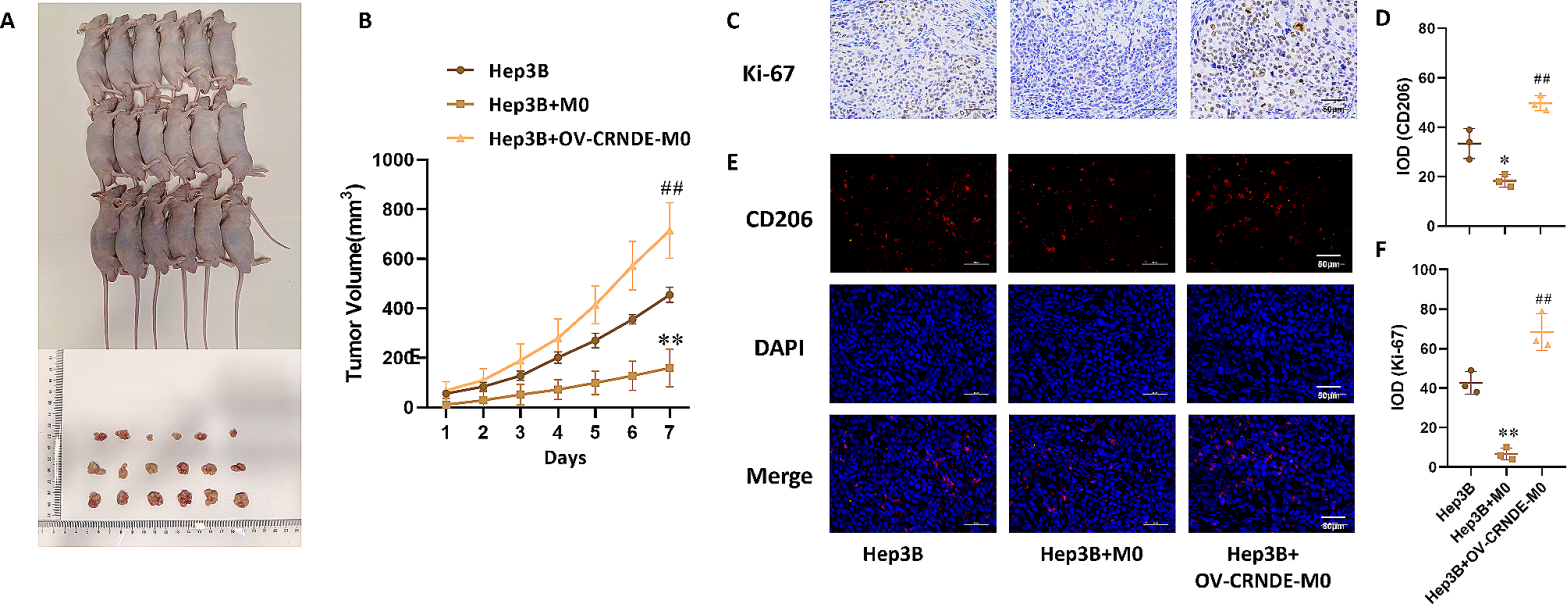
Effects of OV-CRNDE-M0 on the growth of subcutaneous xenograft tumors constructed from Hep3B cells. **A**. The photo of Xenograft model and tumor tissue. **B**. Tumor growth curve was calculated. **C**. Immunohistochemical staining of Ki67 in xenografts tumor tissues. **D**. Quantitative analysis of Immunohistochemical staining of Ki67. **E**. Immunofluorescence staining of CD206 in xenografts tumor tissues. **F**. Quantitative analysis of Immunofluorescence staining of CD206. Compared with Hep3B group, *P < 0.05, **P < 0.01

In order to study the effect of CRNDE in vivo, murine xenograft model was developed via injection of Hep3B cells, Hep3B cells + M0 cells, and Hep3B cells + OV-CRNDE-M0 cells. In comparison to the Hep3B group, tumor volume in Hep3B + M0 group was decreased, while tumor volume in Hep3B + OV-CRNDE-M0 group was increased (*P*<0.05) (Fig. 4A-B). Immunohistochemistry (IHC) results demonstrated that the protein level of Ki67 was downregulated in Hep3B + M0 group compared with Hep3B group, while the protein level of Ki67 was upregulated in Hep3B + OV-CRNDE-M0 group (*P*<0.05) (Fig. 4C and F). In addition, CD206-positve cells were decreased in Hep3B cells + M0 group, which were increased in Hep3B + OV-CRNDE-M0 group (*P*<0.05) (Fig. 4D and E).

### Metabolomic profiling differences among OV-CRNDE-M0, M2, and M0 group

**Fig. 5:**
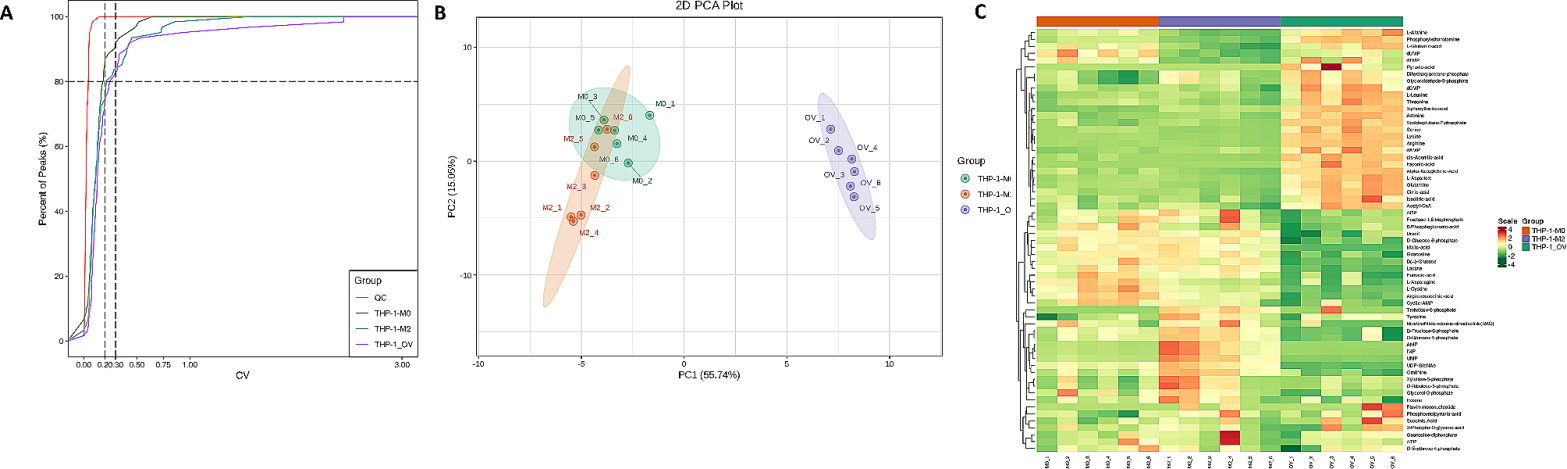
Metabolomics analysis of OV-CRNDE-M0, M0, and M2 groups. The metabolites of M0 cells, M2 cells and OV-CRNDE-M0 cells were detected by LC-MS/MS. **A**. Coefficient of Variation (CV) distribution of samples in each group, abscissa represented the CV value, and the ordinate represents the proportion of the number of metabolites less than the corresponding CV value. **B**. Principal component analysis (PCA) plots of 3 groups, the PC1 and PC2 indicated the first and second principal components, respectively. A single scatter plot represents a sample, and samples from the same group were used in the same color. The distance represents the degree of difference in metabolites. **C**. Heatmap analysis of 61 metabolites expression from the OV-CRNDE-M0, M0, and M2 groups, with the sample name as abscissa, and metabolite as ordinate. Its color represents the level of each metabolite (red color indicates a high level of expression, green color indicates a low level of expression)

To further investigate the effects of CRNDE on macrophages, we profiled the cellular metabolites using LC–MS/MS method. In total, 61 metabolites were identified and quantified from OV-CRNDE-M0, M0, and M2 group. We first explored the stability and reliability of the data, and the proportion of substances with a coefficient of variation (CV) value less than 0.2 in QC samples is higher than 80%, indicating that the instrumental is stability (Fig. 5A). The metabolomic profiles of the 3 groups were then compared by PCA means. PC1 and PC2 contain information about most of the metabolites, with the variance contribution rate 55.74%, and 15.05%, respectively. The distinct separation between M0 and OV-CRNDE-M0 indicated that there were significant differences between the two groups of metabolites, while there were fewer metabolite differences between M2 and M0 group. There was little difference in metabolites seen within the group (Fig. 5B). Furthermore, a heatmap was used to visually represent the alterations in 61 metabolites among OV-CRNDE-M0, M2, and M0 groups. There was no significant difference in the content of different metabolites within the group, there were significant differences in the content of different metabolites among the 3 group (Fig. 5C).

### The differential metabolites among the three groups

**Fig. 6:**
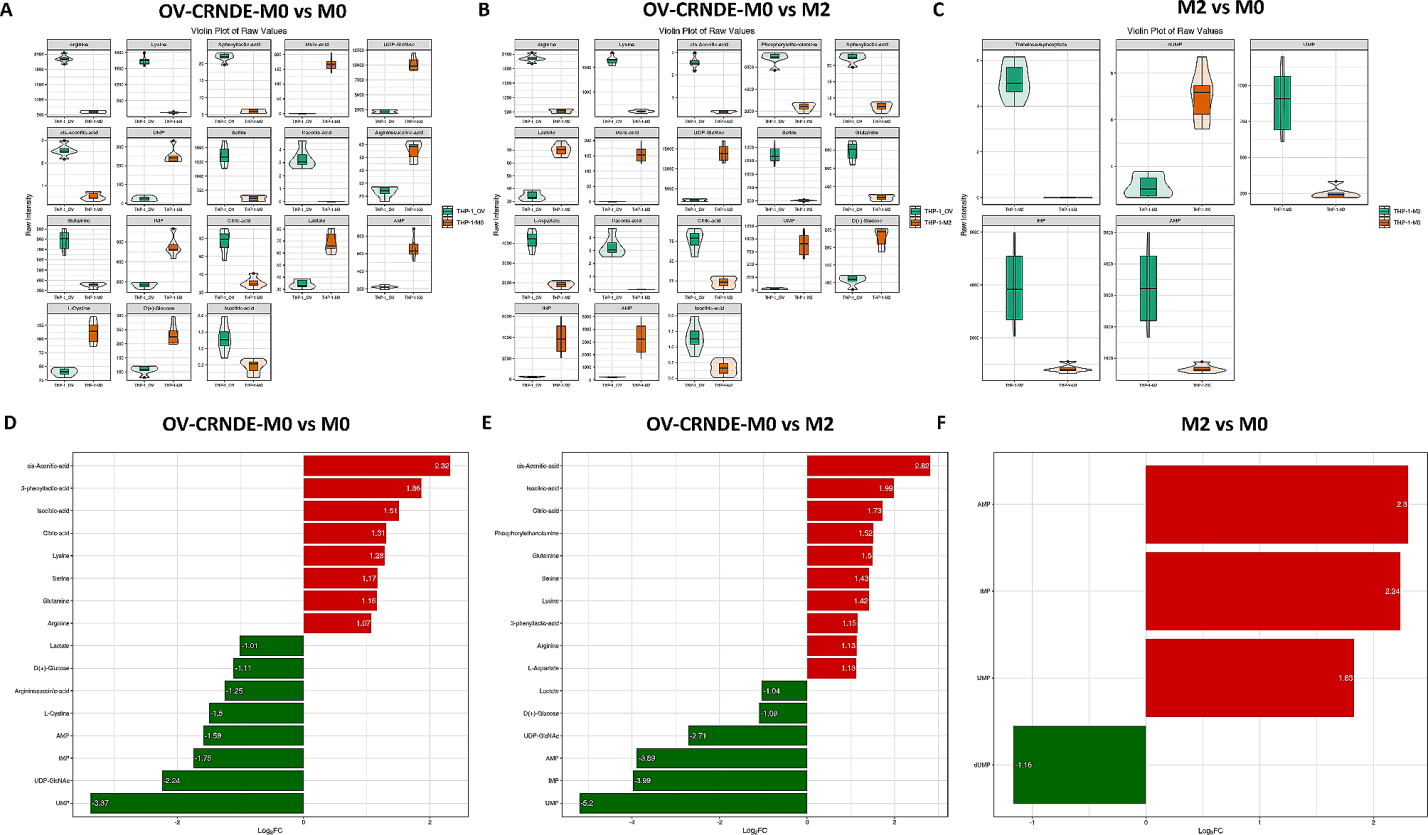
Violin and bar diagram showed significantly altered metabolites among 3 groups. **A**-**C**. Violin diagram showed the differential metabolites between OV-CRNDE-M0 and M0 group (**A**), OV-CRNDE-M0 and M2 group (**B**), M2 and M0 group (**C**). **D**-**F**. Bar diagram showed the fold change of metabolites between OV-CRNDE-M0 and M0 group (**D**), OV-CRNDE-M0 and M2 group (**E**), M2 and M0 group (**F**). The red and green color indicate increased and decreased levels of metabolites

The identified differential metabolites between OV-CRNDE-M0 group and M0 group were shown in Fig. 6A and supplementary materials, with 9 upregulated and 9 downregulated metabolites in OV-CRNDE-M0 group compared to M0 group, including Arginine, Lysine, 3-phenyllactic-acid and so on (Fig. 6A). Of 9 upregulated metabolites, cis-aconitic-acid exhibited the largest increase, 2.32-fold. 3-phenyllactic-acid increased by 1.86-fold, while UMP decrease by 3.34-fold (Fig. 6D). There were 11 upregulated and 7 downregulated metabolites in OV-CRNDE-M0 group compared to M2 group (Fig. 6B), with Cis-aconitic-acid increased by 2.82-fold, UMP decreased by 5.2-fold (Fig. 6E). There were 4 upregulated and 1 downregulated metabolite in M2 group compared to M0 group (Fig. 6C), with AMP increased by 2.3-fold, dUMP decreased by 1.16-fold (Fig. 6F).

### The enrichment and clustering analysis of metabolites among the three groups

**Fig. 7:**
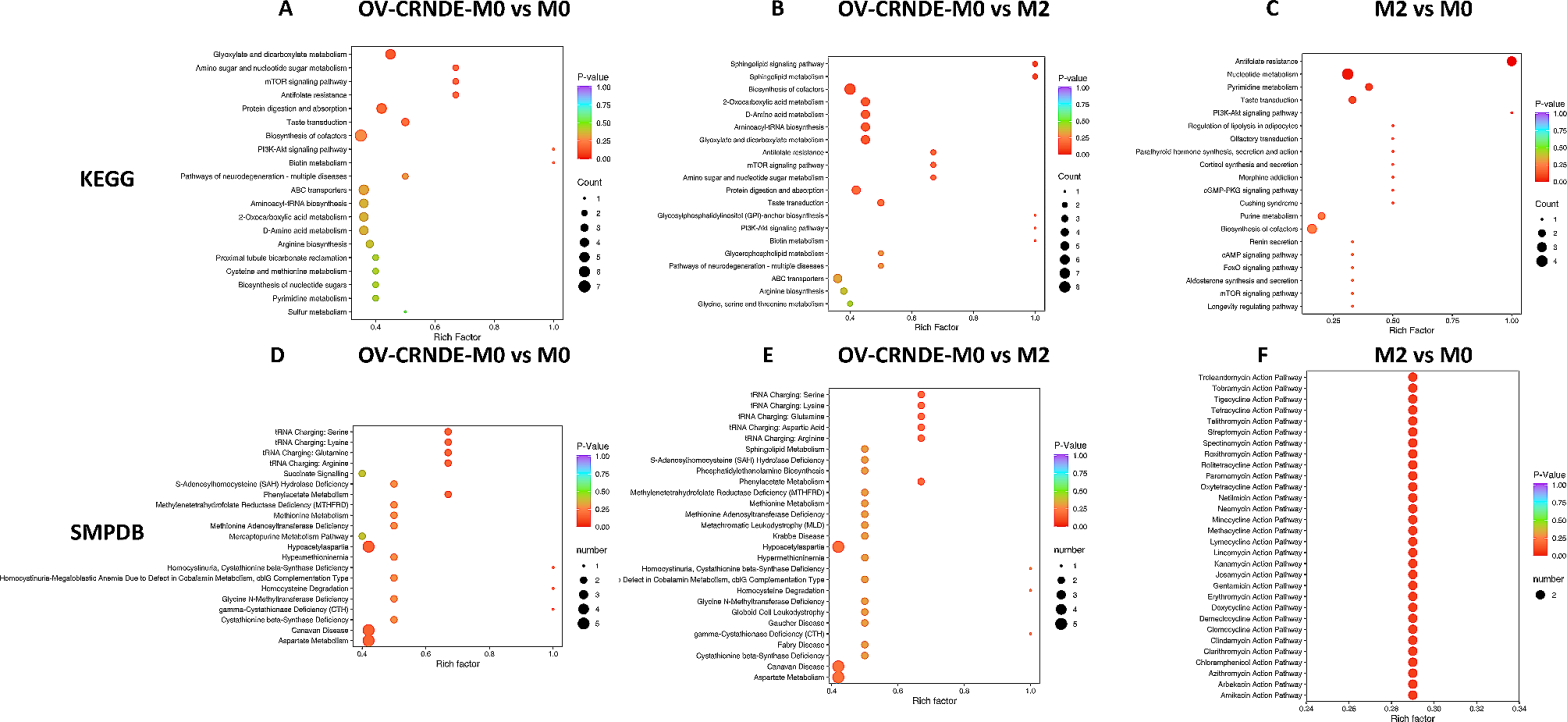
KEGG and SMPDB analysis based on the identified metabolites. **A**-**C**. KEGG enrichment analyses of the identified differentially metabolites between OV-CRNDE-M0 and M0 (**A**), OV-CRNDE-M0 and M2 (**B**), M2 and M0 (**C**). **D**-**F**. SMPDB enrichment map of the identified differential metabolites between OV-CRNDE-M0 and M0 (**D**), OV-CRNDE-M0 and M2 (**E**), M2 and M0 (**F**). The top 20 most significant KEGG terms and top 20 HMDB primary pathways were illustrated, the color was determined by the *P* value, and the size was determined by the number of metabolites in the annotation pathway

The differential metabolites features were enriched to perform the metabolic pathway analysis. KEGG enrichment results revealed that the altered metabolites between OV-CRNDE-M0 group and M0 group were mainly involved in glyoxylate and dicarboxylate metabolism, biosynthesis of cofactors, protein digestion and absorption (Fig. 7A). The altered metabolites between OV-CRNDE-M0 group and M2 group were mainly involved in biosynthesis of cofactors, glyoxylate and dicarboxylate metabolism, protein digestion and absorption (Fig. 7B). Besides, the altered metabolites between M2 group and M0 group were mainly involved in nucleotide metabolism, antifolate resistance, and biosynthesis of cofactors (Fig. 7C). The small molecule pathway database (SMPDB) was used for the corresponding pathway enrichment analysis. Hypoacetylaspartia, Canavan disease, and aspartate metabolism were the significant differential metabolic pathways between OV-CRNDE-M0 group and M0 group (Fig. 7D), which were also the differential metabolic pathways between OV-CRNDE-M0 group and M2 group (Fig. 7E). However, the differential metabolites between M2 group and M0 group were involved in several pathways (Fig. 7F).

**Fig. 8:**
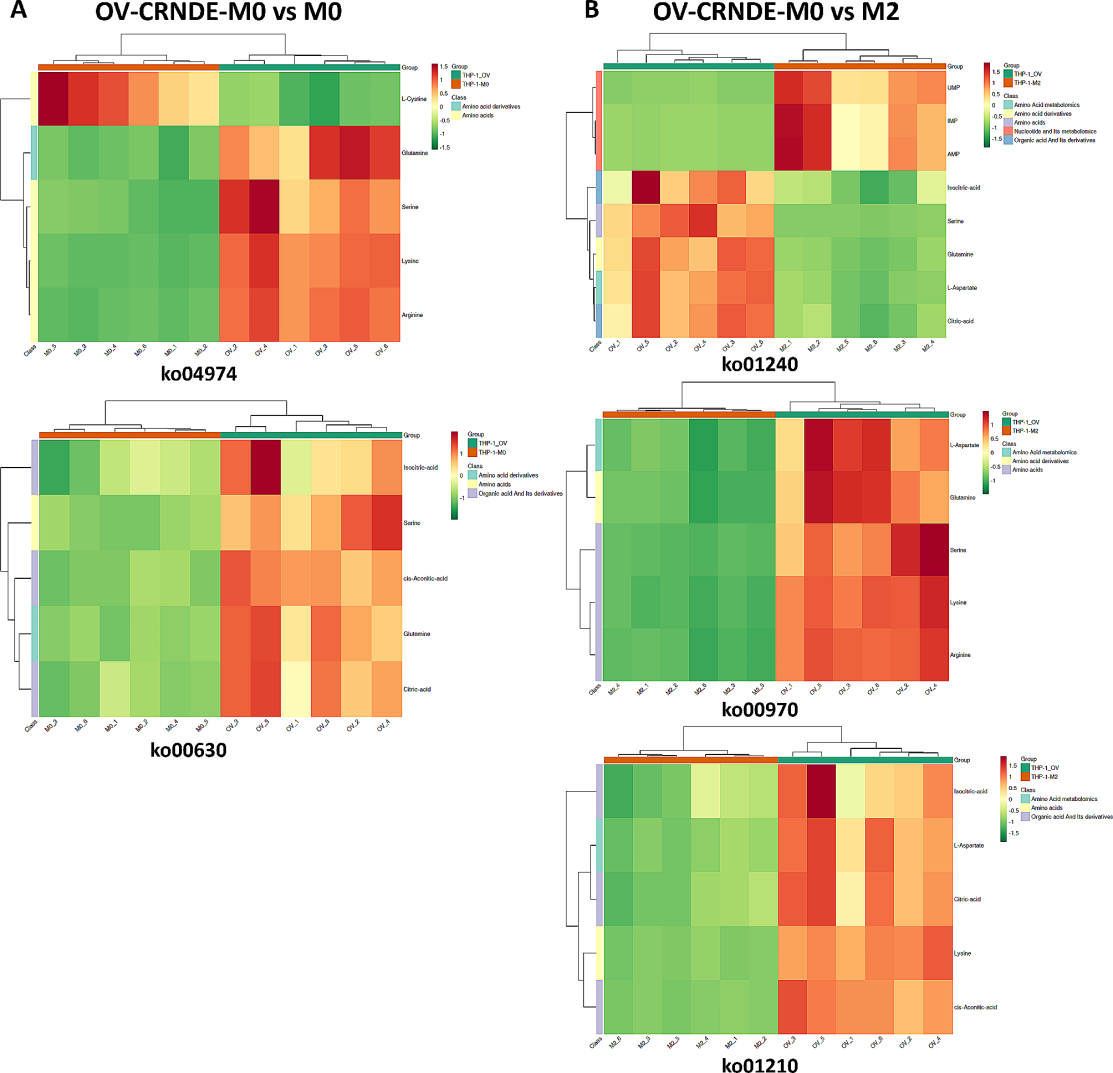
Cluster heatmap of the identified metabolites. **A**. The identified metabolites between OV-CRNDE-M0 and M0 group were classified as amino acids, amino acid derivatives, organic acid and its derivatives. **B**. The identified metabolites between OV-CRNDE-M0 and M2 group were classified as amino acids, amino acid derivatives, organic acid and its derivatives, amino acids metabolomics, nucleotide and its metabolomics

The metabolite levels in OV-CRNDE-M0 group and M0 group were summarized in the cluster heat-maps, where the differential metabolites were classified as amino acid derivatives, amino acid, organic acid and its derivatives (Fig. 8A). The differential metabolites between OV-CRNDE-M0 group and M2 group were classified as amino acid derivatives, amino acid, organic acid and its derivatives, Amino acid metabolomics, nucleotide and its metabolomics (Fig. 8B).

### ERK inhibitor was used to verify the effect of CRNDE on macrophage polarization

**Fig. 9:**
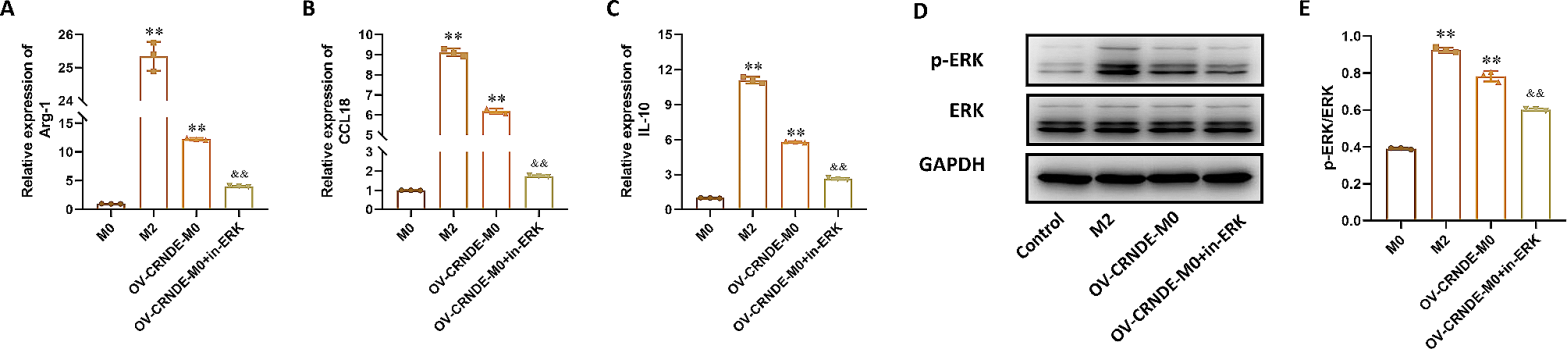
CRNDE could promote M2 macrophage polarization via ERK pathway. **A**-**C**. The relative expression of Arg-1(**A**), CCL-18(**B**), IL-10(**C**) in M0 cell, M2 cells, OV-CRNDE-M0 cells and OV-CRNDE-M0 cells treated with ERK inhibitor. **D**. The protein expression of GAPDH, ERK and p-ERK in M0 cell, M2 cells, OV-CRNDE-M0 cells and OV-CRNDE-M0 cells treated with ERK inhibitor. **E**. The quantitative analysis of Western Blot. Compared with M0 group, *P < 0.05, **P < 0.01

To investigate the correlation between CRNDE promoting macrophage M2 polarization and ERK signaling pathway, OV-CRNDE-M0 were treated with ERK inhibitors. Compared with M0 cells, the relative expression of Arg-1, CCL-18 and IL-10 was increased in M2 cells and OV-CRNDE-M0 cells, which were down-regulated by ERK inhibitor in OV-CRNDE-M0 cells (*P*<0.05) (Fig. 9A-C). Western Blot demonstrated that p-ERK was up-regulated in M2 cells and OV-CRNDE-M0 cells, while ERK inhibitor down-regulated p-ERK level in OV-CRNDE-M0 cells (*P*<0.05) (Fig. 9D-E).

## Discussion

The HCC progression is highly associated with macrophages phenotype in TME, where M2 cells maintain the immunosuppressive and resulted in tumor progression [25]. The underlying mechanism of metabolic reprogramming in macrophages has not been elucidated. CRNDE was reported to regulate cell proliferation and apoptosis in cancers. The specific regulatory mechanisms of CRNDE in macrophage metabolic reprogramming was still unexplored. The results of the research demonstrated that CRNDE overexpression could induce the polarization and metabolic reprogramming of macrophages, thus promoting proliferation, metastasis and invasion of HCC cells.

We first compared the contents of M2 macrophage markers, Arg-1, IL-10, CD163, CD206, and CRNDE in tumor tissues and para-cancerous tissues. These detection indicators were found to be elevated in tumor tissues, CRNDE was also significantly up-regulated in M2 cells compared with M0 cells. Therefore, we hypothesized that CRNDE might be associated with M2 macrophages in tumor. To test this hypothesis, OV-CRNDE-M0 cells were established, and M2 associated genes, Arg-1, CCL-18, and IL-10 were significantly up-regulated in OV-CRNDE-M0 cells, with the upregulation of surface markers CD206. However, these inflammatory factors were downregulated by CRNDE knockdown, which suggesting that CRNDE mediated M2 polarization.

It is generally believed that TAMs communicated with cancer cells directly, where TAMs present M2 phenotype [10]. M2 macrophages are associated with tumor angiogenesis and immune escape by secreting inflammatory cytokines [26]. We co-cultured HCC cells with CM of M0, M2, OV-CRNDE-M0, and sh-CRNDE-M0, the results showed that macrophages overexpressing CRNDE promote the proliferation of both Hep3B cells and Huh-7 cells. However, CRNDE knockdown in macrophages inhibited HCC cells proliferation, migration, and invasion.

The metabolic reprogramming of macrophages seriously affects their function in HCC [10, 29], with the original metabolic pathways, and their intracellular metabolite composition changes significantly. The diversity in metabolite features and their differential regulation reflected metabolic reprogramming. LC-MS/MS-based metabolomics approach is a widely used tool for quantifying metabolites as well as for identifying known and unknown compounds in biological samples [30]. Thus, we used LC–MS/MS to metabolomics to identified significant alterations in macrophage metabolites. The CV diagram showed that the experimental data are very stable. We then performed the PCA before the content difference analysis, which indicating that there was distinct separation among OV-CRNDE-M0 cells, M0 cells, and M2 cells. We examined a total of 68 substances, from which 61 substances were detected. These components can be divided into 11 groups, including nucleotide and its metabolomics, aminos acids, organic acid and its derivatives, phosphate sugars, phosphoric acids, coenzyme and vitamins, amino acid derivatives, amino acid metabolomics, carbohydrate metabolomics, LPE, organic acid and its derivatives. 29.51% (18/61) of the metabolites measured were significantly altered in OV-CRNDE-M0 cells compared to M0 cells.

Cis-aconitic-acid, 3-phenyllactic-acid, isocitric-acid, citric-acid, belonging to the organic acid and its derivatives, were the top 4 metabolites with the largest increase in content. Cis-aconitic-acid, citric-acid, and isocitric-acid were major intermediates in tricarboxylic acid (TCA) cycle [31]. TCA cycle has emerged as the central immunometabolic hub of the macrophage [32], the level of which were elevated in M2 cells [33]. Lactate and glucose were decreased in OV-CRNDE-M0 cells compared to M0 cells. Previously study has demonstrated that glucose was decreased and lactate was increased in M2 cells [33]. Lactate is a metabolite of glycolysis, the level of which reduction indicated that glycolysis in OV-CRNDE-M0 cells was inhibited. Glycolysis is an important way for macrophages to obtain energy, which is crucial for M2 activation [33]. A reduced glycolytic activity in tumor-associated macrophages could favor tumor progression via both nutritional and immunological [34]. Lactate regulated M2 polarization and stimulated expression of M2 polarization markers, including Arg1 [35]. It also promotes tumor growth. Over expression of CRNDE also affects the content of amino acids in metabolites. lysine, serine, and arginine increased by more than 1-fold in OV-CRNDE-M0 cells compared to M0 cells. One of the features describing the macrophages polarization is the change of amino acid metabolism, including arginine metabolism. Arginine is a conditionally essential amino acid whose nutritional requirement is increased during inflammation. Arginine metabolism was significantly altered in M2 macrophages, it can modulate the process of macrophage polarization. Arginine is a major amino acid regulator of macrophage polarization, when its flux into the arginase pathway and generate ornithine to regulate M2 polarization [36]. Seine was the intermediate of TCA, which is required for macrophage IL-1β production [37].

Glutamine was elevated in OV-CRNDE-M0 cells. Glutamine, an amino acid derivative, is metabolized to produce energy through glutaminolysis, which contributes to anaplerotic replenishment of the TCA cycle [38]. The metabolism of glutamine assumes critical importance in HCC proliferation, as the growth of HCC cells escalates the demand for glutamine. This may explain why OV-CRNDE-M0 CM contributed to the proliferation of HCC in vitro and in vivo. Furthermore, a metabolic interplay between glucose and glutamine metabolism existed in the regulation of TAM functions [34]. Furthermore, UMP, IMP, AMP and UDP-GlcNAc decreased in OV-CRNDE-M0 cells compared to M0 cells. These findings showed that overexpression of CRNDE altered metabolites of macrophage significantly. In our enrichment results, overexpression of CRNDE in M0 cells mainly effect biosynthesis of cofactor, glyoxylate and dicarboxylate metabolism, protein digestion and absorption, suggesting a reprogramming of cell metabolism.

ERK1/2 pathway was related to M2 macrophage polarization induced by lactate in breast cancer [27], its role in M2 polarization was also been observed in IL-4-stimulated BM-derived macrophages [28]. These findings were consistent with our results that overexpression of CRNDE induced phosphorylation of ERK, which was inhibited by ERK inhibitor. ERK inhibitor also decreased Arg-1, CCL-18 and IL-10 levels in OV-CRNDE-M0 cells, indicating that CRNDE promoted M2 macrophage polarization via ERK pathway. These in *vitro* results were validated in the HCC model, as the in vivo experiments showed that implantation of Hep3B cells and OV-CRNDE-M0 cells promoted tumor growth and CD206 expression. While implantation of Hep3B cells and M0 cells inhibited tumor growth.

In conclusion, we demonstrated that overexpression of CRNDE in M0 cells contributed to the proliferation and invasion of HCC via ERK pathway, which suggesting that targeting CRNDE may be effective in the treatment of HCC.

## Conclusions

CRNDE regulated the metabolic reprogramming of M2 macrophage via ERK pathway, which thereby contributed to HCC proliferation, migration, and invasion.

## Electronic supplementary material

Below is the link to the electronic supplementary material.

Supplementary Material 1

Supplementary Material 2

Supplementary Material 3

## Data Availability

No datasets were generated or analysed during the current study.

## Declarations

### Supplementary figure

(A) Relative expression of CRNDE in M0 cells following transfection of CRNDE siRNAs. (B) Relative expression of CRNDE in M0 cells transfected with CRNDE overexpression plasmid. Compared with M0 group, **P* < 0.05, ***P* < 0.01.

### Supplementary Information

The identified differential metabolites between OV-CRNDE-M0 group and M0 group, OV-CRNDE-M0 group and M2 group, M2 group and M0 group.
