# Correction: LncRNA CRNDE promotes hepatoma cell proliferation by regulating the metabolic reprogramming of M2 macrophages via ERK pathway

**DOI:** 10.1186/s12935-025-03719-9

**Published:** 2025-03-13

**Authors:** Chao Lin, Tao Jiang, Changyong E, Lun Wang, Tong Chen, Xia Wang, Yien Xiang

**Affiliations:** 1 Hepatobiliary and Pancreatic Surgery, China-Japan Union Hospital of Jilin University, Changchun, China; 2 Gastrointestinal Surgery, Affiliated Hospital of Zunyi Medical University, Zunyi, China; 3 Gastrointestinal Surgery, China-Japan Union Hospital of Jilin University, Changchun, China; 4 General Surgery, China-Japan Union Hospital of Jilin University, Changchun, China; 5 Hepatobiliary and Pancreatic Surgery, the Second Hospital of Jilin University, Changchun, China

**Correction to: Cancer Cell International (2024) 24:193**

10.1186/s12935-024-03380-8

In this article [1], the figures (1–9) were ordered incorrectly. For completeness and transparency, the old incorrect order and the correct order are displayed below.

The original article has been corrected.

Incorrect Fig. 1:


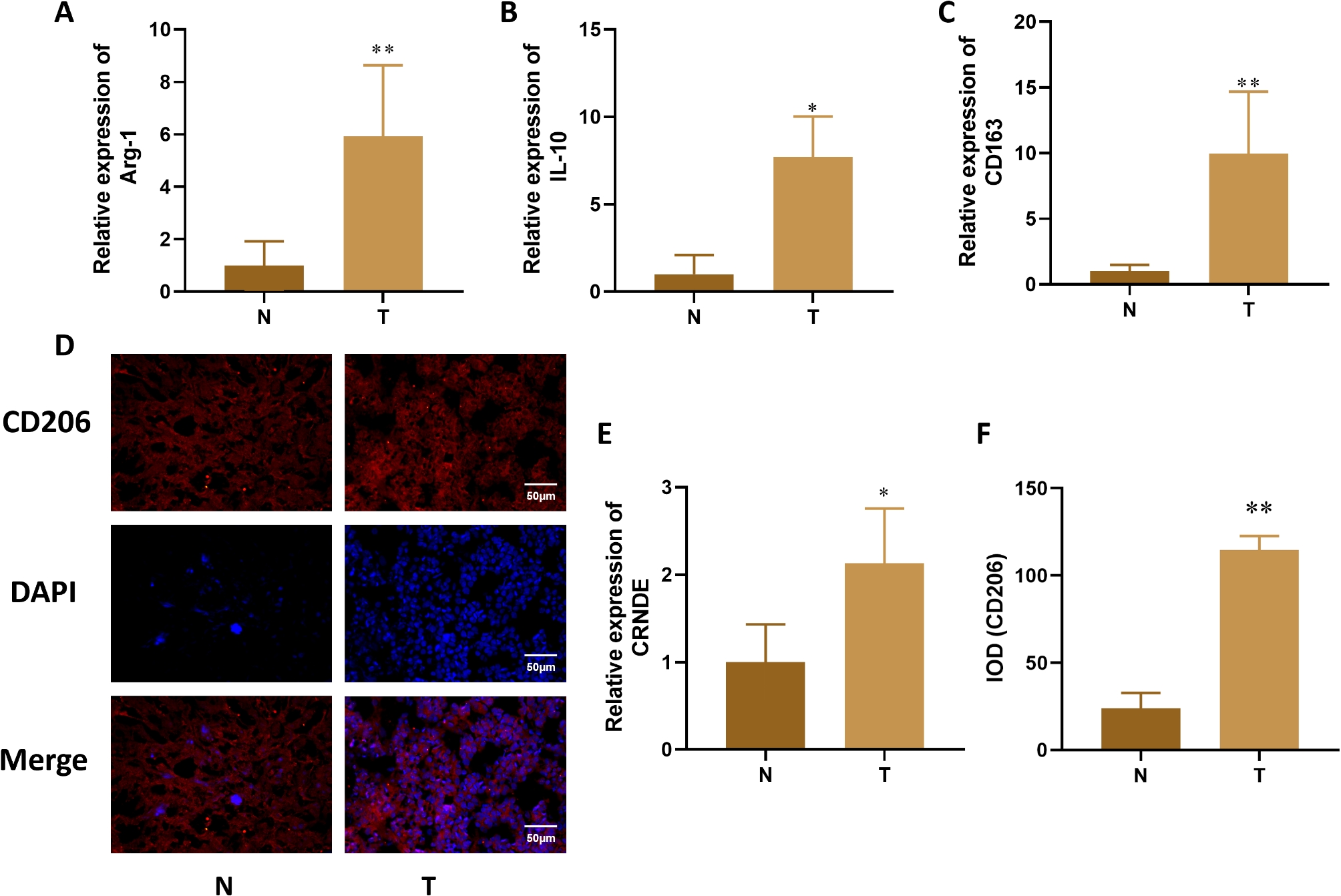


Correct Fig. 1:

**Fig. 1:**
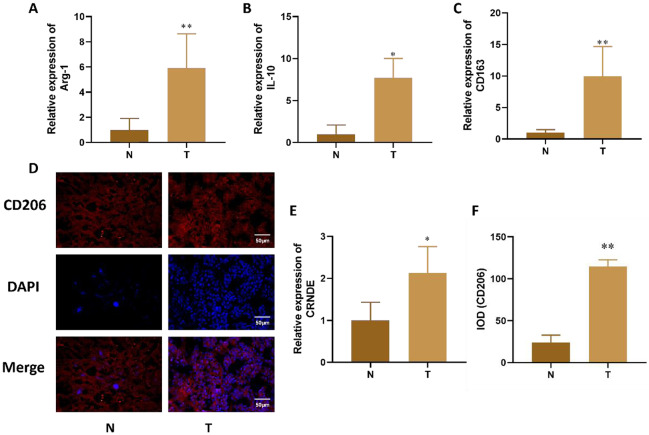
The expression of Arg-1, IL-10, CD163 and CRNDE in liver cancer tissue. **A-C**. Expression changes of M2 macrophage specific markers (Arg-1, IL-10, CD163) detected by qRT PCR in liver cancer tissue. **D.** Immunofluorescence detection of M2 surface antigen (CD206) expression changes in liver cancer tissue. **E.** qPCR was used to detect the expression of CRNDE in tissues. **F.** Quantitative of immunofluorescence staining. Compared with Normal group, *P < 0.05, **P < 0.01

Incorrect Fig. 2:


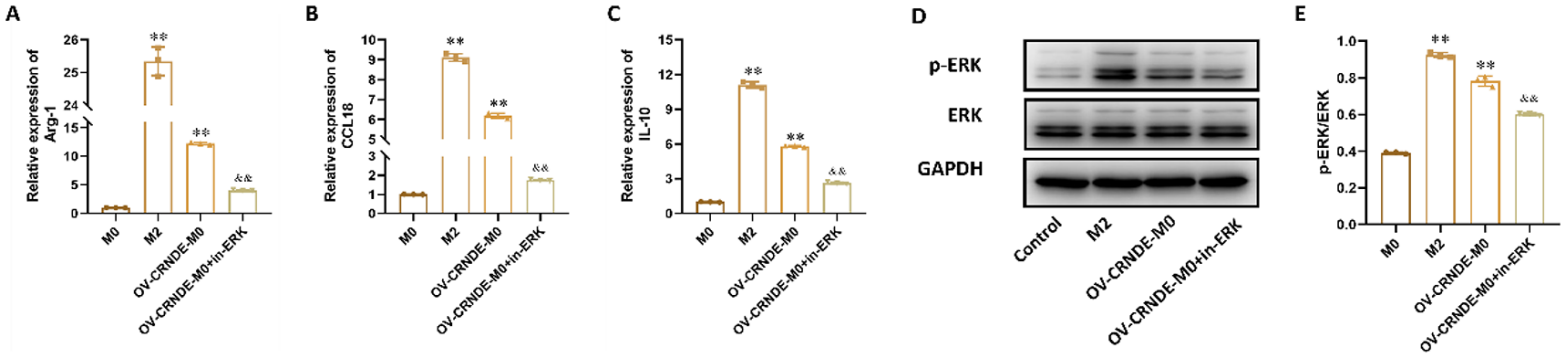


Correct Fig. 2:

**Fig. 2:**
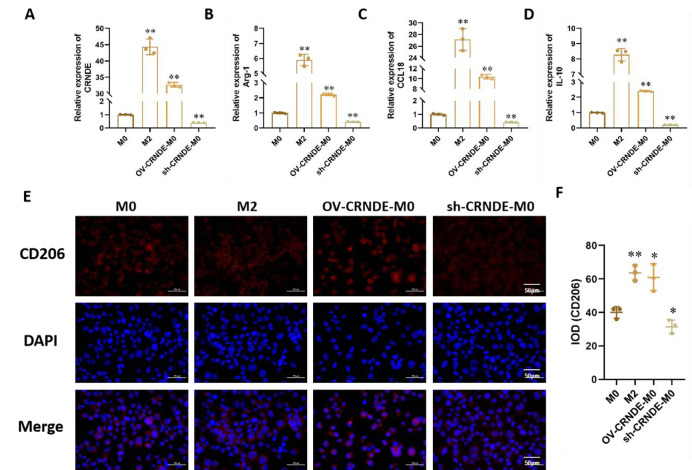
The effect of CRNDE on M2 polarization. **A-D.** Relative expression of CRNDE (**A**), Arg-1(**B**), CCL18 (**C**), IL-10 (**D**) in M0 cells, M2 cells, M0 cells transfected with CRNDE overexpression plasmid (OV-CRNDE-M0), and M0 cells transfected with CRNDE shRNA (sh-CRNDE-M0). **E.** CD206 expression by immunofluorescence staining. **F.** Quantitative of immunofluorescence staining. Compared with M0 group, *P < 0.05, **P < 0.01

Incorrect Fig. 3:


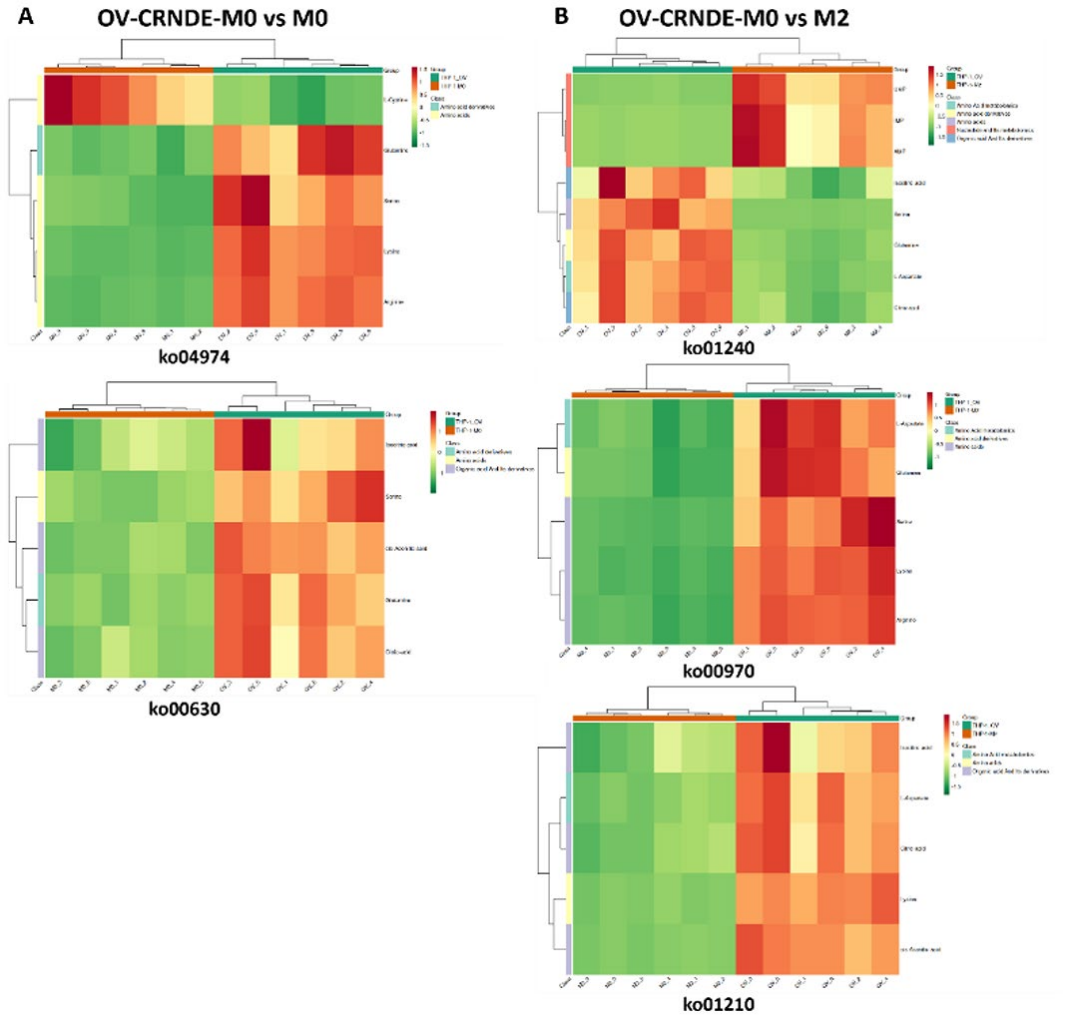


Correct Fig. 3:

**Fig. 3:**
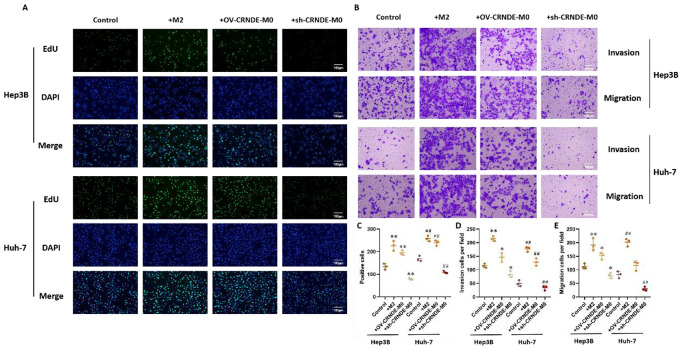
The effect of CRNDE in M0 cells on the proliferation and migration of liver cancer cells. **A.**Cells proliferative property was analyzed by the EdU assays. **B.** The migration/invasion of Hep3B cells and Huh-7 cells were detected by Transwell assay (200×). **C.** The quantitative analysis of EdU assay. **D.** The quantitative analysis of invasion assay. **E.** The quantitative analysis of migration assay. Compared with M0 group, *P < 0.05, **P < 0.01

Incorrect Fig. 4:


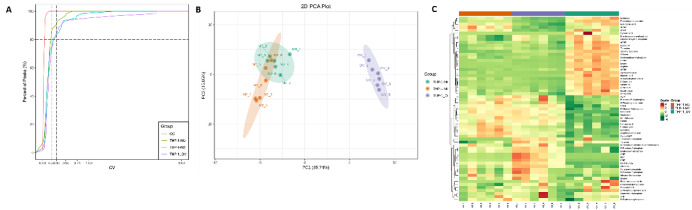


Correct Fig. 4:

**Fig. 4:**
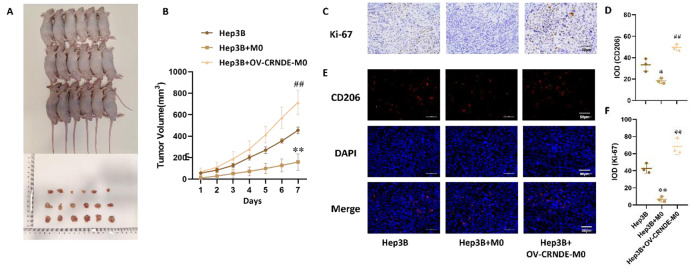
Effects of OV-CRNDE-M0 on the growth of subcutaneous xenograft tumors constructed from Hep3B cells. **A**.The photo of Xenograft model and tumor tissue. **B**. Tumor growth curve was calculated. **C**. Immunohistochemical staining of Ki67 in xenografts tumor tissues. **D**. Quantitative analysis of Immunohistochemical staining of Ki67. E. Immunofluorescence staining of CD206 in xenografts tumor tissues. **F**. Quantitative analysis of Immunofluorescence staining of CD206. Compared with Hep3B group, *P < 0.05, **P < 0.01

Incorrect Fig. 5:


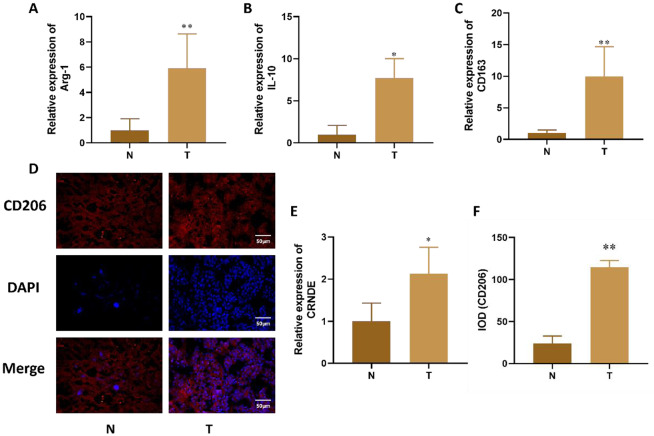


Correct Fig. 5:

**Fig. 5:**
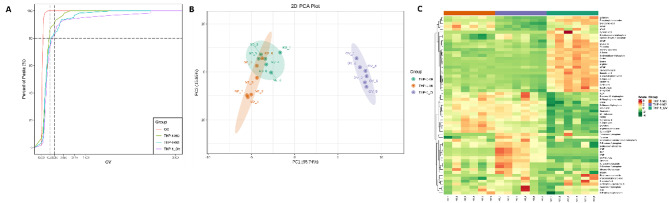
Metabolomics analysis of OV-CRNDE-M0, M0, and M2 groups. The metabolites of M0 cells, M2 cells and OV-CRNDE-M0 cells were detected by LC-MS/MS. **A.** Coefficient of Variation (CV) distribution of samples in each group, abscissa represented the CV value, and the ordinate represents the proportion of the number of metabolites less than the corresponding CV value. **B**. Principal component analysis (PCA) plots of 3 groups, the PC1 and PC2 indicated the first and second principal components, respectively. A single scatter plot represents a sample, and samples from the same group were used in the same color. The distance represents the degree of difference in metabolites. **C.** Heatmap analysis of 61 metabolites expression from the OV-CRNDE-M0, M0, and M2 groups, with the sample name as abscissa, and metabolite as ordinate. Its color represents the level of each metabolite (red color indicates a high level of expression, green color indicates a low level of expression)

Incorrect Fig. 6:


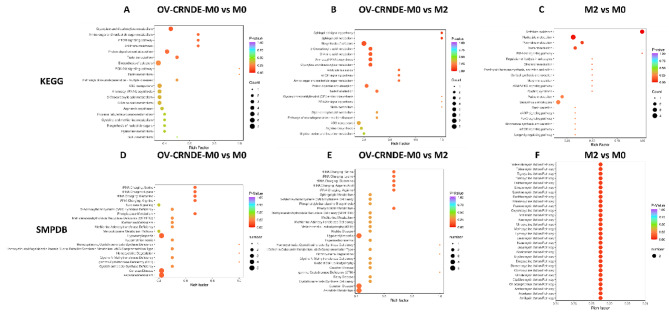


Correct Fig. 6:

**Fig. 6:**
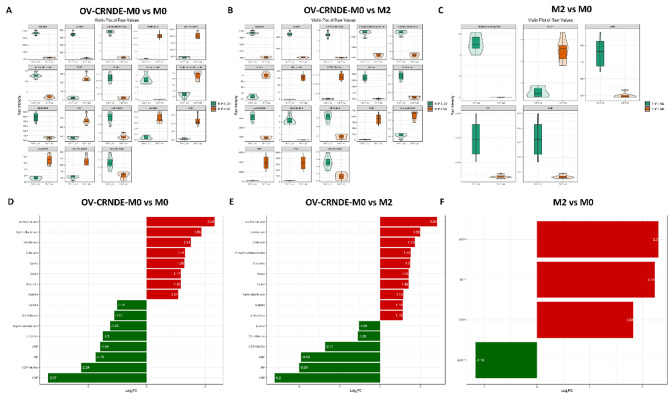
Violin and bar diagram showed significantly altered metabolites among 3 groups. **A-C.** Violin diagram showed the differential metabolites between OV-CRNDE-M0 and M0 group (**A**), OV-CRNDE-M0 and M2 group (**B**), M2 and M0 group (**C**). **D-F.** Bar diagram showed the fold change of metabolites between OV-CRNDE-M0 and M0 group (**D**), OV-CRNDE-M0 and M2 group (**E**), M2 and M0 group (**F**). The red and green color indicate increased and decreased levels of metabolites

Incorrect Fig. 7:


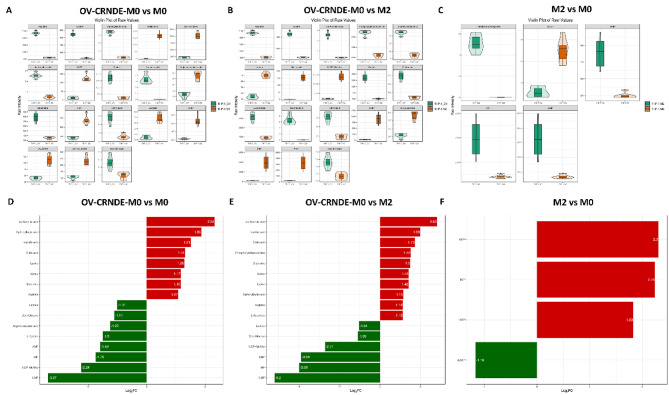


Correct Fig. 7:

**Fig. 7:**
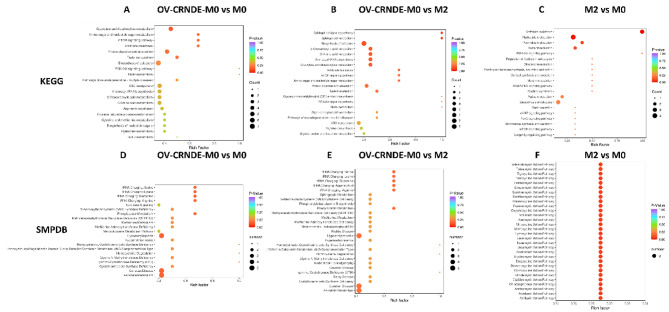
KEGG and SMPDB analysis based on the identified metabolites. **A-C.** KEGG enrichment analyses of the identified differentially metabolites between OV-CRNDE-M0 and M0 (**A**), OV-CRNDE-M0 and M2 (**B**), M2 and M0 (**C**). **D-F.** SMPDB enrichment map of the identified differential metabolites between OV-CRNDE-M0 and M0 (**D**), OV-CRNDE-M0 and M2 (**E**), M2 and M0 (**F**). The top 20 most significant KEGG terms and top 20 HMDB primary pathways were illustrated, the color was determined by the *P* value, and the size was determined by the number of metabolites in the annotation pathway

Incorrect Fig. 8:


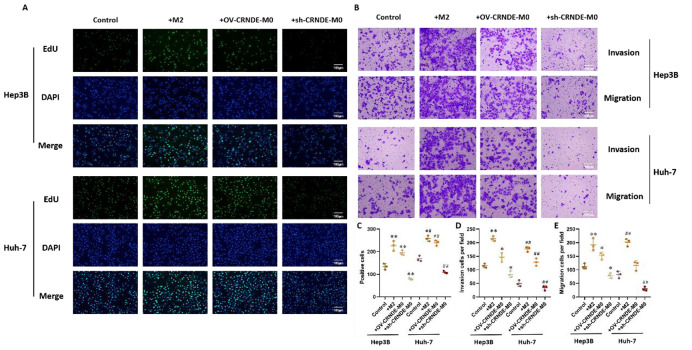


Correct Fig. 8:

**Fig. 8:**
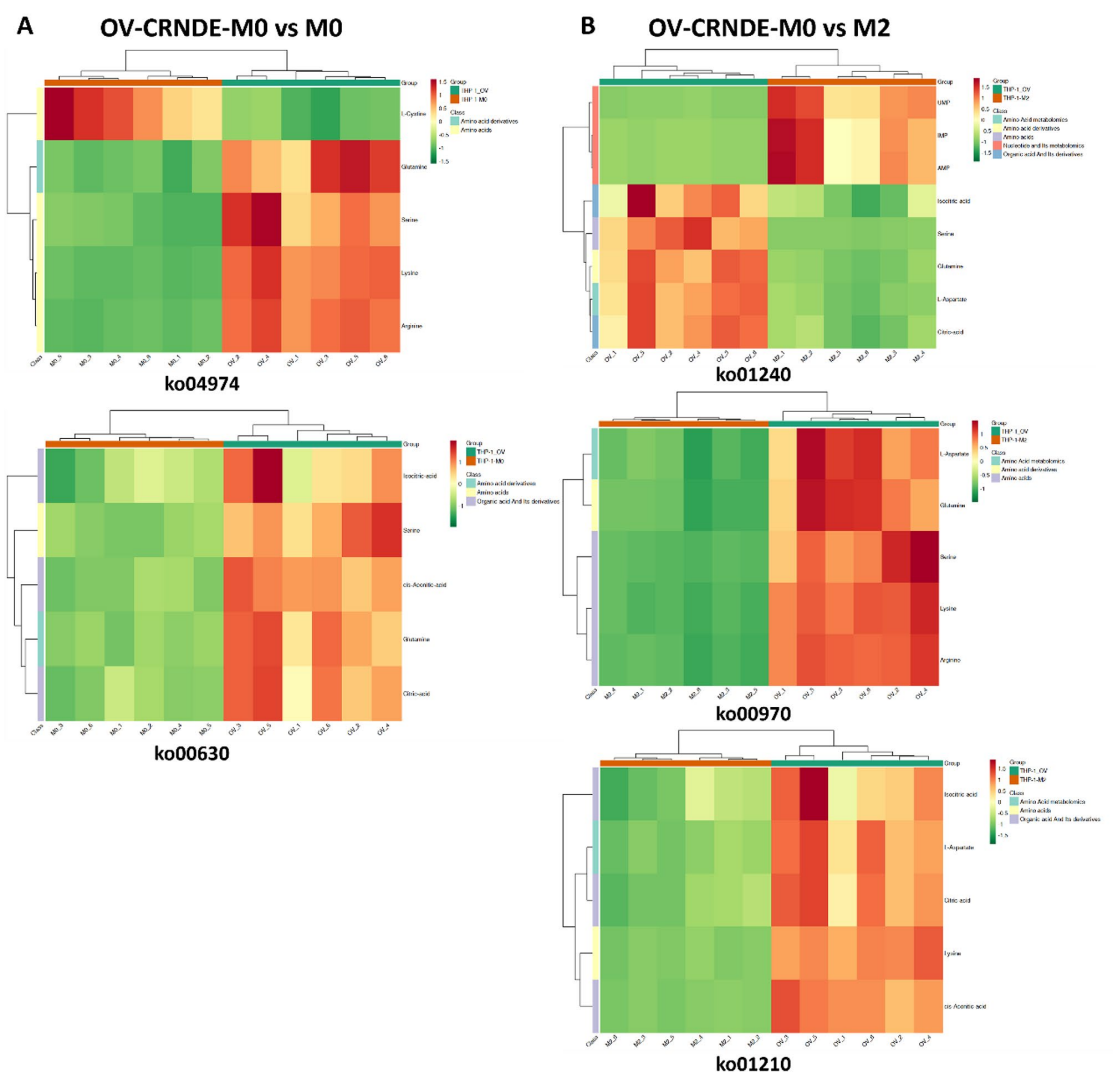
Cluster heatmap of the identified metabolites. **A.**The identified metabolites between OV-CRNDE-M0 and M0 group were classified as amino acids, amino acid derivatives, organic acid and its derivatives. **B.** The identified metabolites between OV-CRNDE-M0 and M2 group were classified as amino acids, amino acid derivatives, organic acid and its derivatives, amino acids metabolomics, nucleotide and its metabolomics

Incorrect Fig. 9:


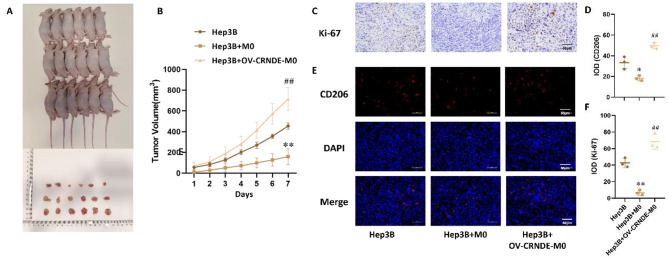


Correct Fig. 9:

**Fig. 9:**
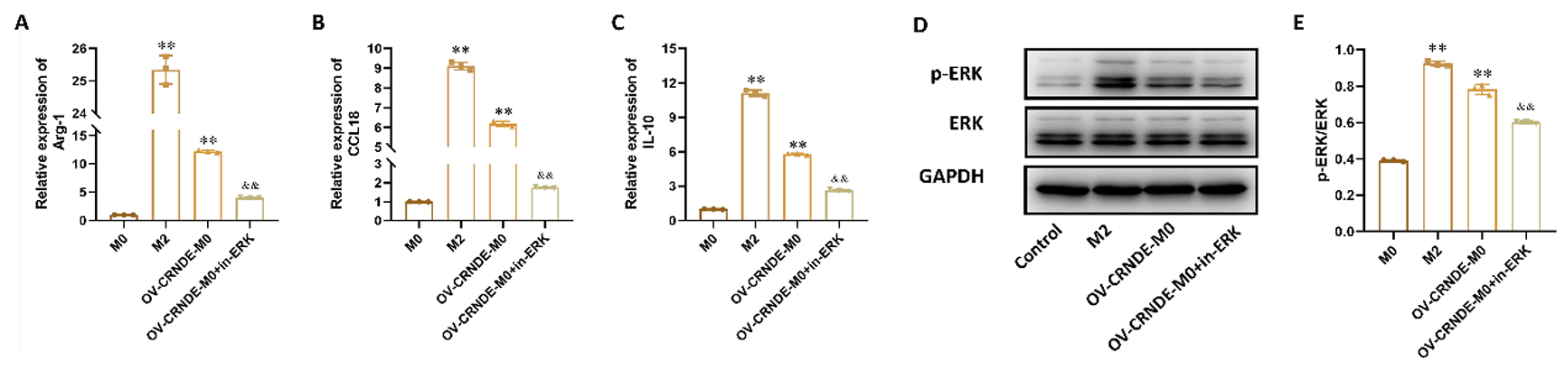
CRNDE could promote M2 macrophage polarization via ERK pathway. **A-C.** The relative expression of Arg-1 (**A**), CCL-18(**B**), IL-10 (**C**) in M0 cell, M2 cells, OV-CRNDE-M0 cells and OV-CRNDE-M0 cells treated with ERK inhibitor. **D.** The protein expression of GAPDH, ERK and p-ERK in M0 cell, M2 cells, OV-CRNDE-M0 cells and OV-CRNDE-M0 cells treated with ERK inhibitor. **E.** The quantitative analysis of Western Blot. Compared with M0 group, *P < 0.05, **P < 0.01
