# Supplementary figures and images for: LncRNA CRNDE promotes hepatoma cell proliferation by regulating the metabolic reprogramming of M2 macrophages via ERK pathway
Source: Cancer Cell Int. 2024 May 31;24:193. doi: 10.1186/s12935-024-03380-8 (PMC11143606; doi:10.1186/s12935-024-03380-8)

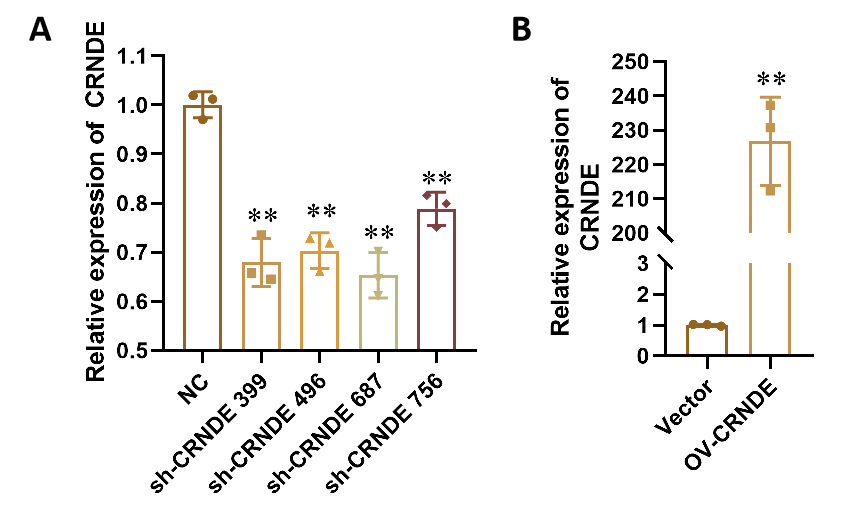

Supplement: Supplementary file 1 — Supplementary Material 1 [file 12935_2024_3380_MOESM1_ESM.tif]
